# Three-dimensional volumetric changes in TMJ space following mandibular advancement surgery in skeletal Class II patients: a CBCT analysis

**DOI:** 10.1590/2177-6709.30.4.e2524300.oar

**Published:** 2025-11-07

**Authors:** Seema GUPTA, Anand V. NIMBAL, Shubham GUPTA, Meghna MITTAL, Chaitra Santoshkumar MASTUD, Jitendra SHARAN

**Affiliations:** 1 Kothiwal Dental College and Research Centre, Department of Orthodontics (Moradabad, Uttar Pradesh, India).; 2 BLDE(DU) Shri B. M. Patil Hospital, Medical College and Research Center, Department of Dentistry (Vijayapura, Karnataka, India).; 3 Dr. D.Y.Patil Dental College and Hospital, Dr. DY Patil Vidyapeeth University, Department of Orthodontics (Pimpri, Pune, India).; 4 All India Institute of Medical Sciences, Department of Orthodontics (Bhubaneshwar, Odisha, India).

**Keywords:** Skeletal Class II, Mandibular advancement, Volumetric, Temporomandibular joint, Space, Classe II esquelética, Avanço mandibular, Volumétrico, Articulação temporomandibular, Espaço

## Abstract

**Objective:**

The aim of the present study was to assess three-dimensional volumetric changes in the temporomandibular joint (TMJ) space after mandibular advancement surgery in skeletal Class II cases with retrognathic mandibles. The null hypothesis for this study posited that there would be no statistical changes after orthognathic surgery.

**Materials and methods:**

A retrospective study was conducted on 30 cone-beam computed tomographic (CBCT) records of skeletal Class II patients with retrognathic mandibles who were treated with bilateral sagittal split osteotomy (BSSO) for mandibular advancement of 6-8 mm. Volumetric assessments of joint space were performed pre-operative (T0), one-week post-operative (T1), and 12-month post-operatively (T2).

**Results:**

The mean anterior joint space exhibited an increase from 612.37 mm^3^ at T0 to 876.67 mm^3^ at T1, followed by a slight reduction to 862.4 mm^3^ at T2. Conversely, the posterior joint space diminished from 861 mm^3^ at T0 to 736.27 mm^3^ at T1 and further to 653.07 mm^3^ at T2. The medial joint space experienced an increase from 525.83 mm^3^ at T0 to 850.93 mm^3^ at T1, before declining to 596.77 mm^3^ at T2. The lateral joint space showed a decrease from 951 mm^3^ at T0 to 762 mm^3^ at T1, subsequently rising to 918.7 mm^3^ at T2. Strong positive correlation coefficients (approaching 1) were observed in the majority of the T1 and T2 comparisons (p < 0.05).

**Conclusion:**

Significant volumetric changes were observed in the TMJ space after mandibular advancement surgery for retrognathism. The changes were maintained even 12 months after surgery, with a slight relapse.

## RESUMO

**Objetivo:**

O objetivo do presente estudo foi avaliar as alterações volumétricas tridimensionais no espaço da articulação temporomandibular (ATM) após cirurgia de avanço mandibular em casos de Classe II esquelética com mandíbulas retrognáticas. A hipótese nula para este estudo foi a de que não haveria alterações estatísticas após a cirurgia ortognática.

**Material e métodos:**

Foi realizado um estudo retrospectivo com 30 documentações contendo tomografia computadorizada de feixe cônico (TCFC) de pacientes com Classe II esquelética com mandíbulas retrognáticas, tratados com osteotomia sagital bilateral (BSSO) para avanço mandibular de 6 a 8 mm. Avaliações volumétricas do espaço articular foram realizadas no pré-operatório (T0), uma semana após a cirurgia (T1) e 12 meses após a cirurgia (T2).

**Resultados:**

O espaço articular anterior médio apresentou um aumento de 612,37 mm3 em T0 a 876,67 mm3 em T1, seguido de uma ligeira redução para 862,4 mm3 em T2. Por outro lado, o espaço articular posterior diminuiu de 861 mm3 em T0 para 736,27 mm3 em T1 e posteriormente para 653,07 mm3 em T2. O espaço articular medial sofreu um aumento de 525,83 mm3 em T0 para 850,93 mm3 em T1, antes de diminuir para 596,77 mm3 em T2. O espaço articular lateral apresentou diminuição de 951 mm3 em T0 para 762 mm3 em T1, aumentando posteriormente para 918,7 mm3 em T2. Coeficientes de correlação fortemente positivos (aproximando-se de 1) foram observados na maioria das comparações T1 e T2 (p < 0,05).

**Conclusão:**

Alterações volumétricas significativas foram observadas no espaço da ATM após cirurgia de avanço mandibular nos casos de retrognatismo. As alterações se mantiveram mesmo 12 meses após a cirurgia, com uma leve recidiva.

## INTRODUCTION

Skeletal Class II malocclusion is a common orthodontic anomaly characterized by a retrognathic mandible, prognathic maxilla, or a combination of both, resulting in a convex facial profile and functional discrepancies. Severe discrepancies are treated by orthognathic surgery, such as mandibular advancement, in cases of retrognathic mandibles.^1^ This condition can profoundly influence facial aesthetics, occlusal relationships, and functionality of the temporomandibular joint (TMJ). Although treatment primarily emphasizes skeletal and dental alignment, ramifications on the TMJ, especially concerning the glenoid fossa and mandibular condyle, are increasingly garnering attention in both clinical practice and scholarly research.^2^ Orthognathic surgery can modify the biomechanical stresses exerted on the TMJ, consequently resulting in remodeling of the glenoid fossa and alterations in the position of the condyle.^3^

Cone-beam computed tomography (CBCT) is an essential modality for diagnostic purposes, surgical strategies, and ongoing patient assessment. Pertaining to the TMJ, this technology facilitates meticulous examination of the osseous structures associated with the joint, thereby enhancing the three-dimensional (3D) evaluation of this anatomical region by providing a more precise delineation of condylar morphology, its potential modifications resulting from adaptive processes, and variations within the joint space.^4^ CBCT is a reliable tool for assessing linear and volumetric measurements of the condyle^5^ and TMJ space.^6^

Ravelo et al.^7^ studied the changes in the condylar position and TMJ space and concluded that significant changes were noticed in the TMJ space; however, no significant changes were observed in the position of the condyle. Condylar instability may lead to a transient relapse, whereas subsequent resorption may result in prolonged skeletal relapse.^8^ Although investigations have been undertaken to evaluate positional alterations of the condyle resulting from orthognathic surgery in patients classified as skeletal Class II with retrognathic mandibles,^9^ there is a notable absence of research focused on volumetric modifications within the TMJ space. Therefore, the aim of the present study was to assess 3D volumetric changes in the TMJ space after mandibular advancement surgery in skeletal Class II cases with retrognathic mandibles. The null hypothesis posited for this study was that there would be no statistical changes after orthognathic surgery.

## MATERIALS AND METHODS

### STUDY DESIGN AND SETTING

This retrospective study was conducted using CBCT records of skeletal Class II patients with retrognathic mandibles who visited the Department of Orthodontics between January 2018 and December 2022. This study was approved by the Institutional Ethics Committee and followed the principles of the Declaration of Helsinki. Written informed consent was obtained from all the patients as a routine procedure of the department to use their records for research purpose, maintaining the confidentiality.

### ELIGIBILITY CRITERIA

Patients with complete records of skeletal Class II with orthognathic maxilla (SNA of 82^0^), retrognathic mandible, and horizontal growth pattern (ANB > 7^0^, SNB < 78^0^, Wits appraisal > 5 mm, SN-GoGn < 27^0^), age > 20 years, irrespective of sex, absence of any TMJ abnormality as verified by clinical and radiological examination, overjet of 7-9 mm, and no degenerative diseases of the condyle, were included in the present study. Those with incomplete records, history of previous orthodontic treatment, TMJ trauma, mandibular asymmetry, congenital abnormalities such as cleft lip and palate or other craniofacial anomalies, and those requiring orthodontic camouflage were excluded from the study.

### SAMPLE SIZE ESTIMATION

A sample size of 30 patients was calculated using G power software, considering 80% power and 5% alpha error. A minimum effect size of 0.46 was considered between pre- and post-orthognathic treatment effects on the TMJ space.^10^

### METHODOLOGY

All subjects in this study underwent pre-operative orthodontic treatment utilizing a non-extraction strategy, conducted by a proficient orthodontist with over 15 years of experience (SG), employing the McLaughlin, Bennett, and Trevisi (MBT) methodology with .022x.028-inch MBT brackets. Each patient was subsequently scheduled for mandibular advancement of 6-8 mm via bilateral sagittal split osteotomy (BSSO) executed by a highly skilled oral surgeon. An intermaxillary splint was utilized to maintain proper occlusion, and rigid internal fixation (RIF) was implemented using a single titanium miniplate on each side secured with 2-3 bicortical 2.0-mm bone screws. CBCT imaging was acquired at T0 (pre-operative), T1 (one-week post-operative), and T2 (12-month post-operatively).

CBCT scans were conducted using a Carestream New Generation CBCT apparatus (Carestream Dental, Atlanta, Georgia) in accordance with a standardized protocol (operating at a voltage of 120 kV, a current of 80 mA, 7-seconds scan time, a field of view (FOV) measuring 10 × 10-cms, a resolution of 0.2 voxels, and 1-mm slice thickness). To achieve a consistent head orientation, the subjects were stabilized with the application of a head stabilizer. During CBCT scanning, the patients were given specific instructions to maintain an erect seated posture with the teeth on maximum intercuspation and to not swallow. To minimize deviations, technician was trained to verify and guide patients into the correct centric occlusal position prior to imaging. This protocol was implemented to ensure that the mandibular position remained stable and comparable between sessions. The eyelids and nasal dorsum were employed as the horizontal and vertical reference axes, respectively, which were established using a laser beam. Image reconstruction for visual analysis was performed using CS imaging software (CS Imaging Software Version 8. Carestream Dental, Atlanta, Georgia, USA). All CBCT scans were obtained with same parameters.

### 3D VOLUMETRIC ANALYSIS OF TMJ SPACE

Slicing was done using Carestream software. CBCT axial plane Digital Imaging and Communications in Medicine (DICOM) files, with a resolution of 429 × 429 pixels at a slice thickness of 300 µm, were imported into the ImageJ software (version 1.54f, Bethesda, Maryland) for analysis. For consistent orientation, all three axes (axial, sagittal, and coronal) were aligned to pass through the center of the TMJ condyle. The landmarks and planes used in this study are listed in Table 1 and were obtained from previous studies.^11^ ^,^ ^12^

**Table 1:** Reference points and planes used in the study.

Reference points and planes	Definition of points and planes
Nasion (N)	Intersection point of frontal bone and nasal bone
Orbitale (Or)	The most inferior point of the bony orbitale (Right: OrR, Left: OrL)
Porion (Po)	The most superior point of the external auditory meatus (Right: PoR, Left: PoL)
Basion (Ba)	Midpoint of the anterior margin of the foramen magnum on the occipital bone
Crista galli (Cg)	The most superior point of the crista galli located in the ethmoid bone
Lateral lip of eminence (Em)	The most inferior and lateral point of articular eminence (Right: EmR, Left: EmL)
Glenoid fossa (Gf)	Thee most superior point of the glenoid fossa (Right: GfR, Left: GfL)
Mid-axial plane (MAP)	A plane passing through OrR, OrL, and PoR
Mid-sagittal plane (MSP)	A plane perpendicular to MAP with passing through Cg and Ba
Coronal plane (COP)	A plane perpendicular to MAP and MSP with passing through Ba
Eminence plane (EmP)	A plane parallel to MAP with passing through Em (Right: EmPR, Left: EmPL)
Glenoid fossa sagittal plane (GfSP)	A plane parallel to MSP with passing through Gf (Right: GfSPR, Left: GfSPL)
Glenoid fossa coronal plane (GfCP)	A plane parallel to COP with passing through Gf (Right: GfCPR, Left: GfCPL)

**Figure 1:**
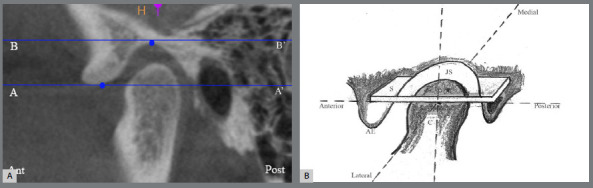
Preparation of slices for TMJ volumetric measurement. **A)** TMJ thickness between two parallel lines (AA’ and BB’) was sliced at slice thickness of 300 µm in axial plane. **B)** Schematic diagram showing slicing (S) in axial plane, where C is condyle, AE is articular eminence, and JS is joint space.

A systematic approach was employed to calculate the volume of the TMJ space. In the axial plane, a line was drawn through the deepest point of the glenoid fossa parallel to the orbital (Or) to the porion (Po) B’ line, which served as the axial plane reference. Another line, AA’ termed the eminence plane, is defined as passing through the lowest point of the articular eminence. The space between these two parallel lines was divided into slices, each 300 µm thick (Fig 1). The area within the boundary of glenoid fossa of each slice was calculated using ImageJ software. The volume of each slice was determined using the following formula: Volume of each slice = slice thickness × area of the slice.

**Figure 2:**
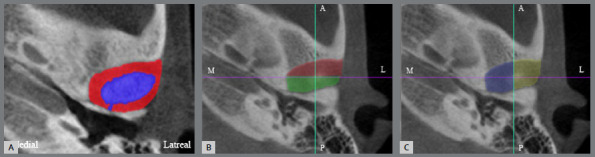
3D volumetric measurement of TMJ space. **A)** Combined volume of TMJ space and condyle (combined red and blue) and TMJ space (only red area). **B)** Division of TMJ space in anteroposterior direction. **C)** Division of TMJ space in mediolateral direction.

The total volume of the space was calculated as the sum of the volumes of all slices between the two parallel lines: volume of the space = ∑n (volume of the slice). The total volume of the TMJ space and condyle was computed by summing the areas of two regions: the red and blue areas in Figure 2A, multiplied by the slice thickness. The TMJ space volume (red area) was then calculated by subtracting the condyle volume (blue area) from the total volume as follows: Volume of TMJ space = [Total area of TMJ space including condyle (Red + Blue) − condylar area (blue)] × slice thickness. To further analyze the TMJ space, it was divided into specific regions. The anterior and posterior joint spaces were delineated by a mediolateral (ML) line parallel to the coronal plane and passing through the condylar center. The anterior space is represented as red and the posterior space as green in Figure 2B. Similarly, the medial and lateral joint spaces were defined using an anteroposterior (AP) line parallel to the sagittal plane, which also passed through the condylar center. The medial space is marked in blue, whereas the lateral space is indicated in yellow (Fig 2C). 

All measurements were performed by the same trained and calibrated evaluator (ShG). The evaluator was calibrated by allowing the evaluator to analyze and perform CBCT test measurements and provide feedback on technique and accuracy. Consequently, an evaluation of ten CBCT scans that were not pertinent to the study for the purpose of independent appraisal was undertaken. The same measurements were repeated on separate occasions by the same evaluator to check the reliability over time. The intra-class correlation (ICC) coefficient value of 0.89 showed high reliability. All measurements were then conducted by same calibrated evaluator who was provided with coded CBCT scans. The images were anonymized and the evaluator was unaware of the patients’ age, sex, and time point of evaluation. Following a washout duration of approximately two weeks, the analysis was repeated on a sample of 30 randomly selected CBCT scans. The ICC value of 0.92 showed high reliability and reproducibility.

### STATISTICAL ANALYSIS

The collected measurements were systematically recorded in a Microsoft Excel spreadsheet and analyzed using SPSS software version 23. The data were assessed for normality using the Shapiro-Wilk test, and the data was found to be normally distributed. Descriptive statistics, including frequency distributions, means, and standard deviations, were utilized for data summarization. To compare the mean differences in TMJ space volume before and after treatment, a repeated measures analysis of variance (ANOVA) was performed, followed by Bonferroni post-hoc analysis. Statistical significance was determined at an alpha level of 0.05. Additionally, correlation analysis was conducted between T1 and T2 to evaluate the effect of treatment on TMJ relapse using Pearson’s correlation. Incomplete records with missing entries and data were excluded from the study.

## RESULTS

**Figure 3:**
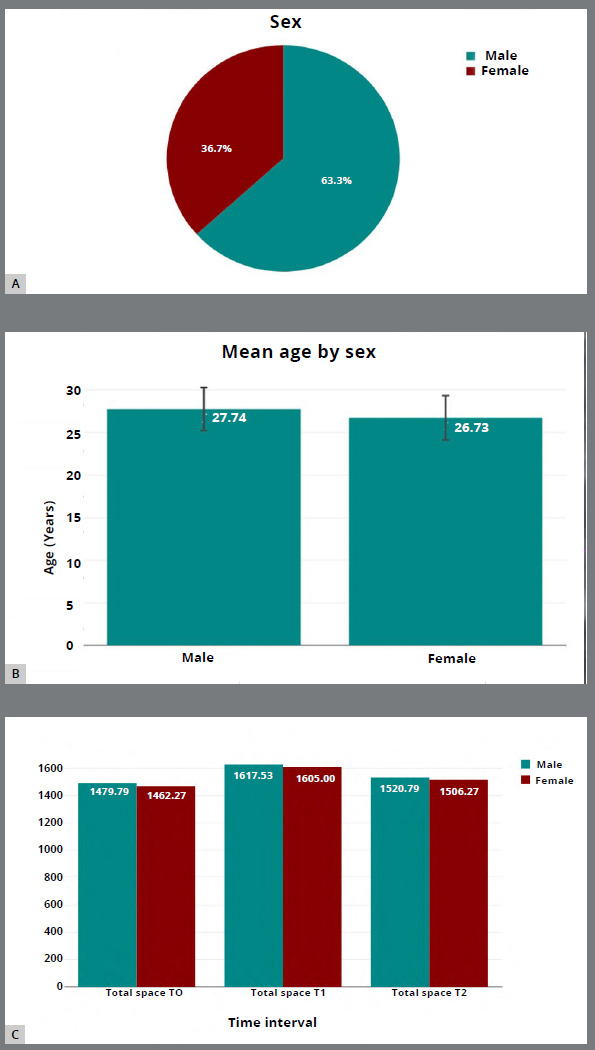
Baseline characteristics of the study sample. **A)** Sex distribution. **B)** Mean age of males and females. **C)** Total TMJ space volume at T0, T1, and T2 in both sexes.

180 records were screened initially and 30 were selected based on the eligibility criteria. The sample consisted of 11 (36.7%) females with a mean age of 26.73 ± 2.6 years, 19 (63.3%) males with a mean age of 27.74 ± 2.53 years. As no statistically significant differences were observed between the right and left sides, the values were averaged for further analysis. The total TMJ space volume was greater in males, compared than in females at all observed time intervals. The total volumetric measurements increased from T0 to T1, and then decreased from T1 to T2 (Fig 3).

Table 2 using the mixed-effects model ANOVA indicated a statistically significant influence of the time interval on the dependent variable (F = 7.71, p = 0.001, η²p = 0.16), signifying a notable effect. Therefore, null hypothesis was rejected in our study. Conversely, neither sex (F = 0.23, p = 0.632, η²p = 0) nor the interaction between sex and time interval (F = 0.00, p = 0.998, η²p = 0) exhibited significant effects. As statistically significant sex differences were not noted, the values were averaged for further analysis.

**Table 2:** Mixed effect Model ANOVA for sex and observed time intervals.

Parameter	Type III Sum of Squares	df	Mean Square	F value	p value	η2p
Sex	4610.95	1	4610.95	0.23	.632	0
Time Interval	307510.16	2	153755.08	7.71	.001*	0.16
Sex x Time Interval	87.93	2	43.97	0	.998	0
Error	1675605.42	84	19947.68			

*p value < 0.05: Significant, df: degree of freedom.

Table 3 depicts the post-hoc Bonferroni analysis revealing statistically significant variances in the total TMJ space volume when comparing T0 and T1 (mean difference: -139.57, p = 0.001) as well as between T1 and T2 (mean difference: 97.47, p = 0.027). Conversely, the comparison between T0 and T2 (mean difference: -42.1, p = 0.751) did not yield statistically significant results.

**Table 3:** Post-hoc Bonferroni test for pairwise comparison of TMJ volume at different time intervals.

Pairwise comparison		Mean Difference	SE	t value	p value
T0	T1	-139.57	36.376	-3.84	0.001*
T0	T2	-42.1	36.376	-1.16	0.751
T1	T2	97.47	36.376	2.68	0.027*

T0: Pre-operative, T1: 1-week post-operative, and T2: 12-month post-operatively, p value < 0.05: Significant, SE: Standard of Error.

Table 4 indicates substantial variations in the TMJ space metrics over the specified timeline (p = 0.001 for all measured parameters). The mean anterior joint space exhibited an increase from 612.37 mm^3^ at T0 to 876.67 mm^3^ at T1, followed by a slight reduction to 862.4 mm^3^ at T2. Conversely, the posterior joint space diminished from 861 mm^3^ at T0 to 736.27 mm^3^ at T1 and further to 653.07 mm^3^ at T2. The medial joint space experienced an increase from 525.83 mm^3^ at T0 to 850.93 mm^3^ at T1, before declining to 596.77 mm^3^ at T2. The lateral joint space showed a decrease from 951 mm^3^ at T0 to 762 mm^3^ at T1, subsequently rising to 918.7 mm^3^ at T2. Post-hoc analysis further revealed that statistically significant differences existed at all time intervals for TMJ space volume except anterior joint space volume between T1 and T2, and lateral joint space volume between T0 and T2 (p > 0.05).

**Table 4:** Comparison of TMJ space volume at different time intervals.

Parameter	Time interval	95% CI for mean	Mean ± SD	p value	Post-hoc analysis
Anterior joint space in mm^3^	T0	587.79 - 636.95	612.37 ± 65.83	0.001*	T0 vs T1 (S)
	T1	846.54 - 906.79	876.67 ± 80.68	T1 vs T2	
	T2	831.35 - 893.45	862.42 ± 83.17	T2 vs T0 (S)	
Posterior joint space in mm^3^	T0	830.88 - 891.12	861.13 ± 80.67	0.001*	T0 vs T1 (S)
	T1	708.35 - 764.19	736.27 ± 74.78	T1 vs T2 (S)	
	T2	628.21 - 677.93	653.07 ± 66.6	T2 vs T0 (S)	
Medial joint space in mm^3^	T0	499.43 - 552.26	525.83 ± 70.79	0.001*	T0 vs T1 (S)
	T1	823.43 - 878.43	850.93 ± 73.65	T1 vs T2 (S)	
	T2	567.13 - 626.42	596.77 ± 79.38	T2 vs T0 (S)	
Lateral joint space in mm^3^	T0	919.78 - 982.22	951.21 ± 83.61	0.001*	T0 vs T1 (S)
	T1	733.68 - 790.32	762.03 ± 75.86	T1 vs T2 (S)	
	T2	890.52 - 946.88	918.72 ± 75.48	T2 vs T0	

T0: Pre-operative, T1: 1-week post-operative, and T2: 12-month post-operatively, p value < 0.05: Significant, SE: Standard of Error, SD: Standard Deviation, CI: Confidence Interval.

Table 5 shows that elevated correlation coefficients (approaching 1) were observed in the majority of T1 and T2 comparisons, signifying that the pre-operative TMJ space volumes were significant predictors of post-operative TMJ space volumes. The presence of p values less than .001 implies that these correlations are statistically significant, reflecting a strong association between T1 and T2 measurements. The persistent positive correlations across all assessments suggested that alterations in TMJ space volumes at T1 were proportionately mirrored at T2. This implies that surgical intervention may have preserved proportional relationships within the joint spaces, despite the overall variations.

**Table 5:** Correlation matrix between volumetric TMJ space measurement at T1 and T2.

TMJ volume	r and p value	Total space volume at T2	Anterior space volume at T2	Posterior space volume at T2	Medial space volume at T2	Lateral space volume at T2
Total space volume at T1	r value	0.98	0.94	0.89	0.88	0.9
	p value	<.001*	<.001*	<.001*	<.001*	<.001*
Anterior space volume at T1	r value	0.83	0.76	0.8	0.76	0.74
	p value	<.001*	<.001*	<.001*	<.001*	<.001*
Posterior space volume at T1	r value	0.92	0.92	0.79	0.81	0.86
	p value	<.001*	<.001*	<.001*	<.001*	<.001*
Medial space volume at T1	r value	0.87	0.79	0.85	0.85	0.72
	p value	<.001*	<.001*	<.001*	<.001*	<.001*
Lateral space volume at T1	r value	0.95	0.95	0.8	0.77	0.94
	p value	<.001*	<.001*	<.001*	<.001*	<.001*

T0: Pre-operative, T1: 1-week post-operative, and T2: 12-month post-operatively, p value < 0.05: Significant, Strong correlation: 0.6≤IrI<0.8; Very Strong correlation: 0.8≤IrI≤1.

## DISCUSSION

This investigation yields significant findings regarding temporal and sex-specific fluctuations in TMJ space volumes. The results highlight the impact of surgical procedures and joint space remodeling processes over time. Subsequently, the results are expounded upon and framed within the current body of literature, and their ramifications are discussed comprehensively.

### SEX DIFFERENCES IN TMJ SPACE VOLUMES

The observed greater TMJ space volume in males than in females aligns with previous studies,^13^ ^,^ ^14^ which highlights that males typically exhibit larger TMJ spaces than females. This difference could be attributed to variations in condylar size, mandibular morphology, and hormonal influences that affect joint development and remodeling.^15^

The absence of significant sex-by-time interaction supports the notion that TMJ space dynamics are more influenced by treatment or time-dependent factors than by baseline anatomical differences between sexes. This finding contrasts with previous studies,^13^ ^,^ ^14^ which could be due to the fact that previous studies have evaluated TMJ space and did not conduct 3D volumetric analysis.

### TEMPORAL CHANGES IN TMJ SPACE VOLUMES

The statistically significant effect of time interval on TMJ space volume indicates that time is a critical determinant in joint space remodeling. The volumes of the anterior and medial joint spaces exhibited an increase from the pre-operative phase to the post-operative phase; however, they subsequently diminished after 12 months. In contrast, the volumes of the posterior and lateral joint spaces showed a decline from pre-operative to post-operative; nevertheless, the lateral joint space volume increased 12 months post-operatively. This pattern could reflect adaptive remodeling aimed at optimizing load distribution across the joint. These joint space volume changes immediately after surgery indicate that the condyle moved posteriorly and laterally, whereas 12 months after surgery, the condyle moved medially, with non-significant changes in lateral space volume between T0 and T2. We were unable to directly compare these results because no prior research has been undertaken to evaluate volumetric alterations in the TMJ space associated with BSSO.

Immediate post-operative changes may not accurately predict long-term outcomes, emphasizing the need for both short- and long-term assessments. Many researchers^16^ ^,^ ^17^ have indicated that individuals with mandibular retrognathism have a higher incidence of condylar resorption after surgery, which leads to condylar remodeling that is often associated with discomfort. This might be due to the fact that significant advancements of the mandible have been documented to elevate the tension within the adjacent soft tissues, resulting in a force directed inferiorly and posteriorly.^18^ This phenomenon induces compressive stresses on the condylar head,^19^ which may precipitate condylar remodeling if the adaptive capacity of the condyle is surpassed. Ravelo et al.^7^ studied changes in the condylar position after mandibular advancement in patients with mandibular retrognathism and observed that after surgery, the condyle moved away from the articular eminence, leading to an increase in the anterior joint space and a decrease in the posterior joint space. This posterior displacement may represent an adaptive mechanism in response to mandibular advancement and alterations in the fulcrum arm of the mandibular structure. Furthermore, since tissues are manipulated during surgical procedures, especially mandibular advancement, subsequent postoperative contraction may result in posterior repositioning of the condyle.^20^

Kim et al.^21^ also observed posterior and lateral displacement of the condyle after mandibular advancement. Harris et al.^22^ observed that the mandibular condyle was displaced posteriorly and medially after 8 weeks of BSSO. This could be due to rotation of the posterior segment with large mandibular advancement. Previous studies of mandibular advancement have observed the medial roll.^23^ ^,^ ^24^ The present study used a RIF with bicortical screws. The implementation of RIF utilizing bicortical screws yields superior stability compared with hybrid and semi-rigid methods. This approach leads to a diminished capacity for condylar adaptation movement and a tendency to revert to pre-operative alignment.^25^ While rigid fixation is distinguished by enhanced stability, it concurrently poses the risk of unintentional condylar torque and displacement throughout the fixation process.^26^

### PREDICTIVE VALUE OF PRE-OPERATIVE TMJ SPACE VOLUMES

The notable correlations between T1 and T2 TMJ space volumes highlight the prognostic significance of pre-operative assessments. This observation indicates that the pre-operative TMJ space volume serves as a dependable predictor of post-operative results. The stability in the proportional relationships within joint spaces following intervention suggests that surgical methods honor and maintain intrinsic anatomical ratios, even in the context of volumetric alterations. Such strong correlations are clinically significant, as they can aid in presurgical planning and prognosis. For instance, understanding the likely trajectory of TMJ space remodeling based on pre-operative data allows clinicians to set realistic expectations and tailor the rehabilitation protocols accordingly. It also reinforces the notion that surgical interventions, when performed with precision, maintain joint architecture integrity.

The present study used CBCT scans for volumetric analysis of the TMJ joint space, which is reliable, and the precision of CBCT in evaluating the dimensions of the TMJ has been substantiated in a contemporary investigation, which determined that the measurements of the joint spaces closely resemble the true anatomical joint spaces.^27^ Our study also showed high reliability and reproducibility of the measurements.

### CLINICAL IMPLICATIONS FOR SURGICAL INTERVENTIONS AND REHABILITATION

The observed dynamics of TMJ space remodeling have direct implications in surgical planning and post-operative care. Surgical interventions that alter TMJ mechanics, such as orthognathic surgery or joint arthroplasty, should consider the adaptive potential of the joint. The findings highlight the significance of monitoring mandibular and TMJ changes in retrognathic patients, particularly those undergoing surgical orthodontic treatment. These findings stress the importance of pre-surgical planning and post-surgical follow-ups to evaluate TMJ health and stability, ensuring optimal treatment outcomes. A thorough pre-surgical assessment should be done using advanced imaging techniques, such as CBCT, to analyze TMJ spaces, condylar positions, and muscle dynamics before surgery, particularly in patients with high risk of condylar resorption. A close communication between orthodontists and maxillofacial surgeons should be fostered to align treatment goals and avoid biomechanical discrepancies. Post-surgical regular follow-ups should be conducted to assess TMJ health and identify potential complications early. Retrognathic patients often exhibit compensatory muscle patterns that can lead to posterior pulling of the mandible during the final phases of surgical orthodontic treatment. Overactivity or imbalance in muscles such as the masseter, temporalis, or suprahyoid group can exacerbate TMJ stress or lead to undesirable mandibular positioning post-surgery. Addressing these muscular dynamics through physical therapy is crucial in ensuring stability and reducing relapse risks.

### LIMITATIONS OF THE STUDY

Although this study provides valuable insights, certain limitations should be acknowledged. The sample size, although adequate for detecting significant effects, may limit the generalizability of the findings. In addition, the retrospective design of our study further affected the generalizability of the findings. The reliance on imaging data for volume measurements, while accurate, does not capture the biochemical and cellular changes that underpin TMJ remodeling. Therefore, future research should aim to include larger and more diverse populations to validate these findings across different demographics, investigate the biochemical and histological correlates of imaging findings to gain a deeper understanding of TMJ remodeling processes, explore long-term outcomes beyond T2 to assess the stability of observed changes, and examine the impact of specific surgical techniques or rehabilitation protocols on TMJ space dynamics in prospective studies. Although the study only evaluated intra-rater agreement which provided confidence that observed changes were due to actual physiological changes and not measurement errors from inconsistent evaluations. However, we acknowledge the clinical importance of inter-examiner reliability. Future studies could incorporate inter-examiner comparisons or leverage AI-based tools for cross-validation of landmark positioning. In this study, we opted to delineate two parallel lines and calculate distances between time points using a standardized formula. This approach was chosen for its simplicity, reproducibility, and the specific nature of our study objectives, which focused on quantifying changes rather than evaluating spatial displacements relative to a superimposed reference. However, incorporating cranial base superimpositions would have provided additional insights into spatial changes and enhanced the robustness of our analyses. Moreover, although efforts were made to standardize bite orientation, we acknowledge that slight deviations in patient cooperation or technician oversight could result in minor differences in mandibular positioning. Therefore, the integration of cranial base superimpositions would allow for the visualization of condylar position relative to a stable anatomical reference. Hence, cranial base superimposition should be incorporated in future research to validate and complement volumetric calculations.

## CONCLUSION

This investigation clarifies the evolving characteristics of TMJ spatial remodeling following BSSO in skeletal class II cases with mandibular retrognathism, emphasizing the substantial role of temporal factors while indicating that sex and its interplay with time are not significant contributors. The volumes of the anterior and medial joint spaces exhibited an increase from the pre-operative phase to the post-operative phase; however, they subsequently diminished after 12 months. In contrast, the volumes of the posterior and lateral joint spaces showed a decline from pre-operative to post-operative; nevertheless, the lateral joint space volume increased 12 months post-operatively. Additional research is essential to expand upon these findings and enhance our understanding of TMJ adaptation and recovery processes.
